# Neuroscience of Emotional Intelligence: An Integrative Narrative Review

**DOI:** 10.7759/cureus.108949

**Published:** 2026-05-16

**Authors:** Arshad Ali, Violet Kulo, Ghaya Al-Rumaihi, Ahsan Sethi, Maha S Elnashar, Nissar Shaikh, Faisal R Jahangiri

**Affiliations:** 1 Clinical Academic Sciences, Qatar University, Doha, QAT; 2 Neurological Sciences, Weill Cornell Medicine, Doha, QAT; 3 Neurosurgery, Hamad General Hospital, Doha, QAT; 4 Health Professions Education, University of Maryland, Baltimore, Baltimore, USA; 5 Neurosurgery, Hamad Medical Corporation, Doha, QAT; 6 Department of Public Health, College of Health Sciences, Qatar University, Doha, QAT; 7 Department of Population Health Sciences, Weill Cornell Medicine Qatar, Doha, QAT; 8 Surgical Intensive Care, Hamad Medical Corporation, Doha, QAT; 9 Neurology, NeuroCare.AI Academy, Dallas, USA; 10 Neurophysiology, Global Innervation LLC, Dallas, USA; 11 Department of Neuroscience, School of Behavioral and Brain Sciences, University of Texas at Dallas, Richardson, USA

**Keywords:** emotional intelligence, emotions, empathy, neural networks, neuroscience, social cognition

## Abstract

Emotional intelligence (EI) has emerged as a critical neurocognitive construct linking affective processing, social behavior, and adaptive functioning. This integrative narrative review synthesizes multidisciplinary findings to map the biological underpinnings of EI across genetic, epigenetic, neuroanatomical, and neurophysiological domains. Drawing on evidence from neuroimaging, molecular genetics, neurochemistry, and brain connectivity studies, the review suggests EI as a product of dynamic interactions between prefrontal-limbic circuits, neurotransmitter systems, and environmentally sensitive regulatory mechanisms. Key brain structures, including the prefrontal cortex, amygdala, anterior cingulate cortex, and insula, operate within coordinated networks that support the recognition, regulation, and social cognition of emotions. Neurotransmitters such as dopamine, serotonin, oxytocin, and gamma-aminobutyric acid (GABA) modulate the emotional reactivity and cognitive control essential to EI. Epigenetic modifications further explain the lifelong plasticity of emotional capacities in response to experience. Anchored in the process model of emotion regulation and the social brain hypothesis, this review provides a cohesive neuroscientific framework for EI. It outlines its translational implications in education, healthcare, and affective computing. By consolidating current advances, this review may help inform precision interventions and policy initiatives aimed at enhancing emotional resilience, empathy, and psychological well-being.

## Introduction and background

Emotional intelligence (EI) is the capacity to perceive, use, understand, and manage emotions. It has gained substantial recognition as a core component of human cognition, interpersonal functioning, and psychological well-being. Salovey and Mayer [1] initially conceptualized EI as a distinct form of intelligence, distinct from traditional cognitive assessments, highlighting emotional and social competencies. However, it is essential to acknowledge that the study of EI is rooted in David Wechsler’s 1940 notion of the “non-collective aspect of general intelligence” [2]. The theoretical landscape of EI has evolved through three dominant models: the ability model, the trait model, and the mixed model. The ability model frames EI as a measurable cognitive capacity related to emotion-focused reasoning and problem-solving [3]. It includes two principal domains: 1) experiential EI, which refers to the capacity to perceive emotional signals and use them to facilitate cognitive processes, and 2) strategic EI, which includes the capacity to comprehend intricate emotional states and manage emotions adaptively.

In contrast, the trait model, developed by Petrides and Furnham [4], interprets EI as a constellation of personality traits linked to emotional self-perception. It consists of 15 elements categorized into four higher-order factors: self-actualization, sociability, well-being, and self-control. The mixed model integrates abilities with motivational and interpersonal factors. There are two mixed models, one created by Goleman [5], which comprises five EI skills: self-awareness, self-regulation, motivation, empathy, and social skills. The second mixed model, the Bar-On model of emotional-social intelligence, shall consist of five components: intrapersonal skills, interpersonal skills, adaptability, stress management, and general mood [6]. Collectively, these frameworks have informed diverse empirical investigations and laid the groundwork for examining the neural, genetic, and developmental underpinnings of emotional intelligence within the context of cognitive neuroscience and behavioral health. Traditionally studied through psychological frameworks, EI is increasingly viewed as a neurocognitive construct supported by distinct and interacting neural systems [7,8]. Ability EI is primarily associated with dynamic neural processing in the prefrontal-limbic circuitry. In contrast, trait EI reflects more stable patterns of connectivity associated with personality-related structures, such as the default mode network. This mapping highlights distinct neurocognitive pathways underlying different EI constructs [8].

Building on these psychological models, emerging evidence from neuroscience suggests that emotional intelligence extends beyond a purely behavioral or personality-based construct and is supported by dynamic neurocognitive systems involved in emotional processing, social interaction, and self-regulation. Rather than functioning as an isolated psychological trait, EI appears to arise from the coordinated activity of interconnected brain regions shaped by both genetic predispositions and environmental experiences. This integrative narrative review explores the neuroscientific foundations of EI by examining how brain structure, neural connectivity, neurophysiology, molecular genetics, and developmental plasticity collectively contribute to emotional perception, regulation, and adaptive social behavior. Evidence from neuroimaging, neurophysiology, and molecular studies increasingly supports the view that EI represents a biologically grounded and developmentally shaped construct that integrates cognitive and affective processes. To strengthen this conceptual framework, the review incorporates the Process Model of Emotion Regulation, which explains how higher-order cognitive systems modulate emotional responses through prefrontal-limbic interactions, alongside the Social Brain Hypothesis, which proposes that human brain evolution was driven in part by the demands of increasingly complex social environments [9,10]. Together, these perspectives provide a clearer bridge between traditional psychological theories of EI and their underlying neurobiological mechanisms. Understanding EI through a neuroscience-informed perspective may help guide the development of more targeted educational, clinical, and organizational interventions aimed at enhancing emotional regulation, empathy, resilience, and socially adaptive functioning.

## Review

Methodology

A comprehensive literature search was conducted across five databases (Embase, Scopus, Medline, PsycINFO, and Web of Science) from inception through October 31, 2025. Search strategies included Medical Subject Headings (MeSH) and relevant keywords to capture the multidimensional scope of EI and its neurobiological underpinnings. The search syntax was adapted to match each database's indexing structure, ensuring methodological consistency. Additionally, the reference lists of the included studies were manually reviewed to identify relevant articles that the database searches had missed. Priority was given to peer-reviewed studies with methodological rigor, systematic reviews or meta-analyses, and seminal theoretical works relevant to the neurobiological foundations of EI. Particular emphasis was placed on studies employing objective neuroscientific approaches, including neuroimaging, neurophysiology, and molecular or genetic methodologies (see Appendices A and B for the search strategy and inclusion/exclusion criteria). An initial yield of 6,330 records was screened, and 355 full-text articles were assessed against predefined criteria. Ultimately, 90 peer-reviewed studies were included in the synthesis of the neurobiological foundations of EI. As this study was designed as an integrative narrative review, no formal quantitative synthesis or meta-analysis was performed. Findings were synthesized descriptively and interpreted through a multidisciplinary conceptual framework focused on the neuroscience of emotional intelligence.

The rationale for this review of the neuroscience of EI at this juncture is rooted in the recent proliferation of high-resolution neuroimaging studies, advances in molecular genetics, and a deeper understanding of neurodevelopmental plasticity, all of which demand an integrated perspective. Moreover, the growing emphasis on mental health, emotional resilience, and prosocial behavior in response to global challenges such as the COVID-19 pandemic underscores the need for neuroscience-informed strategies to foster EI throughout the lifespan. While previous reviews have often approached EI from behavioral or psychological angles, only a few have thoroughly explored its neural correlates, genetic foundations, and developmental potential within a single, cohesive framework [11,12].

Results

The results of the narrative review synthesize multidisciplinary, integrative evidence on the biological underpinnings of EI across seven domains. These include: (1) genetic and epigenetic influences, highlighting heritability and environmental modulation; (2) evolutionary and embryological origins of emotion-related brain systems; (3) anatomical substrates such as the prefrontal cortex, amygdala, and insula; (4) histological specializations supporting emotional processing; (5) neural network integration across cortical and subcortical regions; (6) neurotransmitter systems regulating affective states; and (7) neurophysiological mechanisms that mediate dynamic emotion-cognition interactions. Together, these findings support the interpretation of EI as a neurocognitive construct shaped by both inherited and experience-dependent processes.

Genetics and epigenetics

Research in behavioral genetics has consistently shown that EI, particularly trait EI, exhibits moderate heritability. Twin and family studies estimate that genetic factors account for about 30% to 50% of individual differences in EI [13]. These heritable components are thought to underlie emotional awareness, regulation, and interpersonal sensitivity. Genetic correlation analyses further reveal that EI shares variance with broader psychological constructs, including the general factor of personality and key personality traits such as neuroticism, agreeableness, and extraversion [14]. These findings suggest that the genetic determinants of EI are not isolated but rather part of a broader genetic architecture influencing socioemotional behavior and affective dispositions. Notably, pleiotropy, in which a single gene affects multiple traits, has been proposed as a mechanism underlying the overlap between EI and other personality trait constructs [15]. For example, a study using the Trait Emotional Intelligence Questionnaire (TEIQue) found that trait EI and humor styles shared significant genetic overlap, indicating common genetic factors that modulate emotional expressivity and interpersonal behavior [14,16]. Similarly, polymorphisms in genes related to serotonin (5-HTTLPR), dopamine (DRD4, COMT), and oxytocin (OXTR) pathways have been associated with aspects of empathy, emotional regulation, and social bonding, traits central to EI [17-19].

Beyond static genetic inheritance, epigenetic mechanisms provide a robust framework for understanding how life experiences shape EI. Epigenetics involves reversible molecular changes, including DNA methylation, histone acetylation, and microRNA activity, that modify gene expression without altering the DNA sequence [20-22]. These mechanisms serve as an interface between the genome and the environment, mediating the impact of social experiences, stress, and early-life adversity on emotional development. Early caregiving quality, exposure to trauma, and chronic psychosocial stress are among the most influential environmental inputs that modulate epigenetic signatures [22]. For instance, alterations in the methylation patterns of glucocorticoid receptor genes (e.g., NR3C1) and oxytocin receptor genes (OXTR) have been linked to dysregulation of the hypothalamic-pituitary-adrenal (HPA) axis and deficits in emotional regulation and attachment behavior [23,24]. These findings underscore the biological embedding of emotional experiences and explain how environmental adversity can disrupt the neuroendocrine and neurochemical systems involved in EI. Importantly, epigenetic processes also account for the plasticity of EI across the lifespan. Longitudinal studies suggest that positive social environments, such as supportive relationships and emotionally enriching experiences, can reverse adverse epigenetic modifications, enhance neural connectivity, and strengthen emotional competencies [25]. Neuroplastic adaptations in emotion-related brain circuits, particularly in the prefrontal cortex, amygdala, and anterior cingulate cortex, may be mediated by these epigenetic shifts [26].

Thus, EI arises from a dynamic interplay between inherited predispositions and environmentally responsive molecular changes. This integrative model not only accounts for individual variability in emotional skills but also offers insight into how interventions, such as mindfulness training, trauma-informed care, and emotion-focused therapy, can reshape the neurobiological underpinnings of EI through experience-dependent gene expression. As such, understanding the genetic and epigenetic architecture of EI opens new pathways for precision approaches to enhancing emotional well-being, resilience, and social competence.

Evolutionary and embryological development

The origins of EI are deeply rooted in evolutionary biology and neurodevelopmental processes that have shaped the social brain. From an evolutionary perspective, the development of emotional systems has conferred adaptive advantages, particularly for species reliant on cooperation, caregiving, and group living [27]. Basic affective responses are not uniquely human; many non-human animals exhibit emotion-like behaviors that facilitate survival through social signaling, territoriality, and threat detection. Cabanac [28] famously described “emotional fever” in reptiles, a phenomenon in which stress triggers transient thermoregulatory changes, suggesting that emotional responses predate neocortical evolution and are deeply conserved across vertebrate lineages. At the neural level, the development of the limbic system, including the amygdala, hippocampus, hypothalamus, and cingulate cortex, represents a key evolutionary advancement. This system, situated around the brainstem and responsible for managing primitive vegetative functions, enhances the processing of emotionally significant stimuli and the regulation of physiological responses [29]. These structures form the foundation of the mammalian emotional brain, facilitating rapid threat appraisal, social bonding, and affiliative behaviors. MacLean’s [30] triune brain model, although now considered an oversimplification, helped underscore the hierarchical layering of affective and cognitive systems that evolved. The expansion of the neocortex, especially in primates, enabled more advanced regulation of limbic functions and laid the groundwork for a “rational brain” with sophisticated emotional competencies, such as empathy, theory of mind, and moral reasoning [31].

Embryologically, the systems governing EI arise from the telencephalon, a derivative of the embryonic forebrain. The sequential maturation of emotional brain circuits reflects both ontogeny and phylogeny [32]. Subcortical regions, such as the amygdala and hypothalamus, differentiate and become functional earlier in gestation, supporting primary affective reactivity, including expressions of distress or pleasure in neonates [33]. In contrast, the prefrontal cortex, including regions involved in emotional regulation such as the ventromedial prefrontal cortex (vmPFC), undergoes protracted development into early adulthood, reflecting its role in the nuanced control of emotion, decision-making, and social behavior [34]. The timing of these developmental processes has profound implications. For example, during critical periods of brain plasticity, environmental inputs can shape the architecture and connectivity of emotion-related regions, with lasting consequences for emotional competencies. Notably, the structural complexity of areas such as the anterior cingulate cortex (ACC) and the insula has increased over evolutionary time [31]. These areas, rich in von Economo neurons, are implicated in rapid emotional judgment, empathy, and social sensitivity, which are critical to EI [35]. Their emergence in large-brained mammals, including great apes and humans, supports the notion that EI is an evolutionary adaptation to increasingly complex social environments [36].

In summary, EI reflects a layered biological heritage that integrates ancient subcortical emotional systems with newer cortical regions responsible for conscious regulation and social cognition. Its embryological emergence and evolutionary refinement underscore its fundamental role in shaping adaptive human behavior.

Anatomical structures

EI is backed by an intricate network of brain areas that work together to support the perception, understanding, regulation, and expression of emotions. This neural architecture integrates cognitive and affective systems. It includes both cortical and subcortical structures, as well as white matter tracts that mediate their connectivity [37]. The prefrontal cortex (PFC) is central to EI, particularly the ventromedial (vmPFC), dorsolateral (dlPFC), and orbitofrontal cortex (OFC). The vmPFC is implicated in value-based decision-making, emotional regulation, and social reasoning, functions essential to managing emotions in oneself and others [38]. The dlPFC contributes to cognitive control and working memory, playing a role in reappraisal and other emotion regulation strategies [38,39]. The OFC integrates affective information to guide behavior in social contexts, influencing empathy, moral judgment, and reward processing [40].

The anterior cingulate cortex (ACC) serves as a conflict monitor, playing a crucial role in detecting emotional salience and regulating emotional responses [41]. It connects extensively with both limbic and prefrontal regions, making it critical for integrating emotional and cognitive information [42]. Meanwhile, the insula, particularly the anterior insula, is associated with interoceptive awareness, the emotional processing of internal bodily states, and the integration of these states with affective stimuli [43,44]. Subcortical structures, including the amygdala and hippocampus, are also fundamental to EI. The amygdala detects emotionally salient stimuli, especially those related to fear and threat, and triggers autonomic responses [45]. It also interacts with the hippocampus, which supports the formation and contextualization of emotional memories [46-48]. White matter pathways facilitate structural and functional connectivity among these regions. The uncinate fasciculus, which connects the OFC with the anterior temporal lobe and the amygdala, supports the retrieval of emotion-laden memories and emotional learning [49]. The corpus callosum and superior longitudinal fasciculus facilitate interhemispheric and frontoparietal communication, which is essential for emotional self-regulation and executive function [50,51].

Collectively, this network of cortical and subcortical structures underpins the core domains of EI, enabling individuals to interpret emotional signals, modulate their emotional responses, and navigate complex social interactions effectively. As such, the neuroanatomy of EI provides a foundational understanding of interventions in education, mental health, and leadership development.

Histological architecture

Neocortical layers, with pyramidal neurons in layers I, II, and V, support cortico-cortical and subcortical integration. Dense dendritic spines enhance synaptic complexity, while inhibitory interneurons in layers II and IV modulate excitatory input to maintain emotional balance [52]. In the ACC, which includes granular and dysgranular regions, layer V contains spindle-shaped and pyramidal neurons that project to limbic and brainstem areas, enabling integration of visceral signals with autonomic and behavioral responses [53]. The anterior insula, transitioning from agranular to granular cortex, supports interoceptive integration and higher-order emotional awareness. Its abundance of von Economo neurons (VENs) in layer V suggests specialization in rapid emotional appraisal and social cognition, a feature it shares with the ACC [35,54]. The amygdala consists of histologically distinct nuclei: the basolateral complex, rich in glutamatergic pyramidal-like neurons, projects to the PFC and hippocampus, while the GABAergic central nucleus regulates autonomic arousal [55,56]. The hippocampus exhibits a trilaminar architecture, with pyramidal cells in the CA regions and granule cells in the dentate gyrus. LTP in this context supports the consolidation of emotional memory, and affective states modulate neurogenesis [57,58]. White matter tracts such as the uncinate fasciculus and cingulum bundle consist of myelinated axons whose structural integrity, reliant on oligodendrocyte health, is essential for effective emotion regulation [59].

In summary, EI-related regions exhibit specialized cellular architecture, including VENs, layered pyramidal networks, and a dynamic excitatory-inhibitory balance, forming the histological basis for adaptive emotional and social behavior.

Neural networking and connection

The neural architecture underlying EI comprises interconnected cortical and subcortical regions that collectively process, regulate, and interpret emotional stimuli [51]. These structures are organized into functionally specialized networks linked through white-matter tracts and dynamic neural signaling pathways [60]. Central to this system is the interaction among the prefrontal cortex (PFC), the limbic system, and large-scale functional networks, including the salience and default mode networks [51,52]. The vmPFC, dlPFC, and OFC coordinate higher-order cognitive processes involved in emotion regulation, inhibitory control, and moral reasoning [61]. These regions maintain reciprocal connections with subcortical structures such as the amygdala and hippocampus, forming the neural substrate for emotional appraisal and contextualization [55,62]. The amygdala, a major limbic hub, detects emotionally salient stimuli and transmits signals to the PFC, initiating regulatory feedback mechanisms that align affective responses with contextual demands [45,63,64]. The ACC serves as an integrative hub for conflict monitoring, attentional control, and emotional salience detection. It connects extensively with both the executive control and salience networks, contributing to performance monitoring and adaptive emotional regulation [64,65]. Similarly, the anterior insula plays a central role in interoceptive awareness and empathy by linking bodily states with emotional cognition [66,67]. Its close functional connections with the amygdala and ACC facilitate real-time awareness of internal affective states [62].

Structural connectivity among these regions is mediated by white matter tracts such as the uncinate fasciculus, superior longitudinal fasciculus, and cingulum bundle [49]. These pathways enable coordinated transmission of emotional and cognitive information across frontal, temporal, and limbic regions [68]. For example, the uncinate fasciculus links the OFC with the anterior temporal lobe and amygdala, supporting the integration of emotional memory with decision-making processes [66,69]. Functional neuroimaging studies further demonstrate that EI depends on coordinated activity across large-scale brain networks [70,71]. The salience network, anchored by the anterior insula and ACC, identifies emotionally significant stimuli and facilitates dynamic switching between the default mode network (DMN) and the central executive network [51,72,73]. This network coordination is essential for attentional modulation, emotional control, and adaptive social functioning. Recent spectral dynamic causal modeling (spDCM) studies also demonstrate directed connectivity between right- and left-hemispheric PFC regions that correlates with emotion regulation performance, highlighting the importance of effective interhemispheric communication in EI [74].

Collectively, these interconnected neural systems provide the structural and functional foundation for emotional perception, regulation, empathy, and adaptive social behavior.

Neurobiology (neurotransmitters)

While large-scale neural networks provide the structural framework for EI, neurotransmitter systems regulate the dynamic signaling processes that enable emotional perception, motivation, social behavior, and emotion regulation. These neurochemical systems modulate communication within prefrontal-limbic circuits, influencing both emotional reactivity and adaptive emotional control [75].

Dopamine plays a central role in reward processing, motivation, executive function, and reinforcement learning, all of which contribute to emotionally intelligent behavior. Dopaminergic pathways, particularly the mesocorticolimbic circuit, project from the ventral tegmental area (VTA) to the nucleus accumbens and PFC [76]. Through these pathways, dopamine influences goal-directed behavior, attentional control, working memory, and emotional decision-making [77]. Serotonin (5-HT) is critically involved in mood regulation, impulse control, and social behavior. Serotonergic projections from the raphe nuclei contribute to emotional stability, empathy, and social cooperation, whereas reduced serotonin activity is associated with emotional dysregulation and heightened emotional reactivity [78]. Polymorphisms in the serotonin transporter gene (5-HTTLPR) have also been linked to individual differences in emotional regulation and affective responsiveness [79,80].

Oxytocin, synthesized in the hypothalamus and released by the posterior pituitary, is strongly associated with empathy, trust, attachment, and prosocial behavior. By modulating amygdala and PFC activity, oxytocin enhances emotion recognition and interpersonal sensitivity [81]. Increasing evidence suggests that oxytocinergic pathways contribute significantly to emotional understanding and social connectedness, both central domains of EI [82]. Norepinephrine, released primarily from the locus coeruleus, regulates emotional arousal, vigilance, attention, and stress responses. Through its interactions with amygdala-hippocampal circuitry, norepinephrine influences the encoding and retrieval of emotionally salient memories [83]. Although acute elevations may enhance alertness, chronic activation can impair emotional regulation and resilience [84]. Gamma-aminobutyric acid (GABA), the principal inhibitory neurotransmitter in the brain, contributes to emotional stability by regulating excitatory signaling within prefrontal and limbic circuits. Enhanced GABAergic activity has been associated with reduced anxiety and improved emotional control [85,86]. In contrast, glutamate, the primary excitatory neurotransmitter, is essential for synaptic plasticity, learning, and emotional memory formation, particularly within amygdala-hippocampal pathways [87]. Dysregulated glutamatergic signaling has been implicated in affective instability and mood disorders, underscoring its importance in emotional processing [88].

Together, these neurotransmitter systems dynamically regulate the neural circuitry underlying EI, shaping how emotions are perceived, interpreted, and regulated across interpersonal and intrapersonal contexts.

Neurophysiological processing

Recognizing, understanding, controlling, and using emotions is an essential part of EI and is deeply embedded in neurophysiological processes that balance emotional reactivity with cognitive control [89]. This neural interplay enables individuals to respond adaptively to emotional stimuli, manage stress, empathize with others, and sustain effective social relationships. Central to this system is the limbic network, particularly the amygdala, which functions as the brain’s emotional alarm. When emotionally salient stimuli are detected, the amygdala rapidly evaluates sensory input and can trigger a stress response before the neocortex engages [90]. This rapid, bottom-up activation, termed “amygdala hijack” by Daniel Goleman (5), occurs when emotional intensity overwhelms rational judgment.

**Figure 1:**
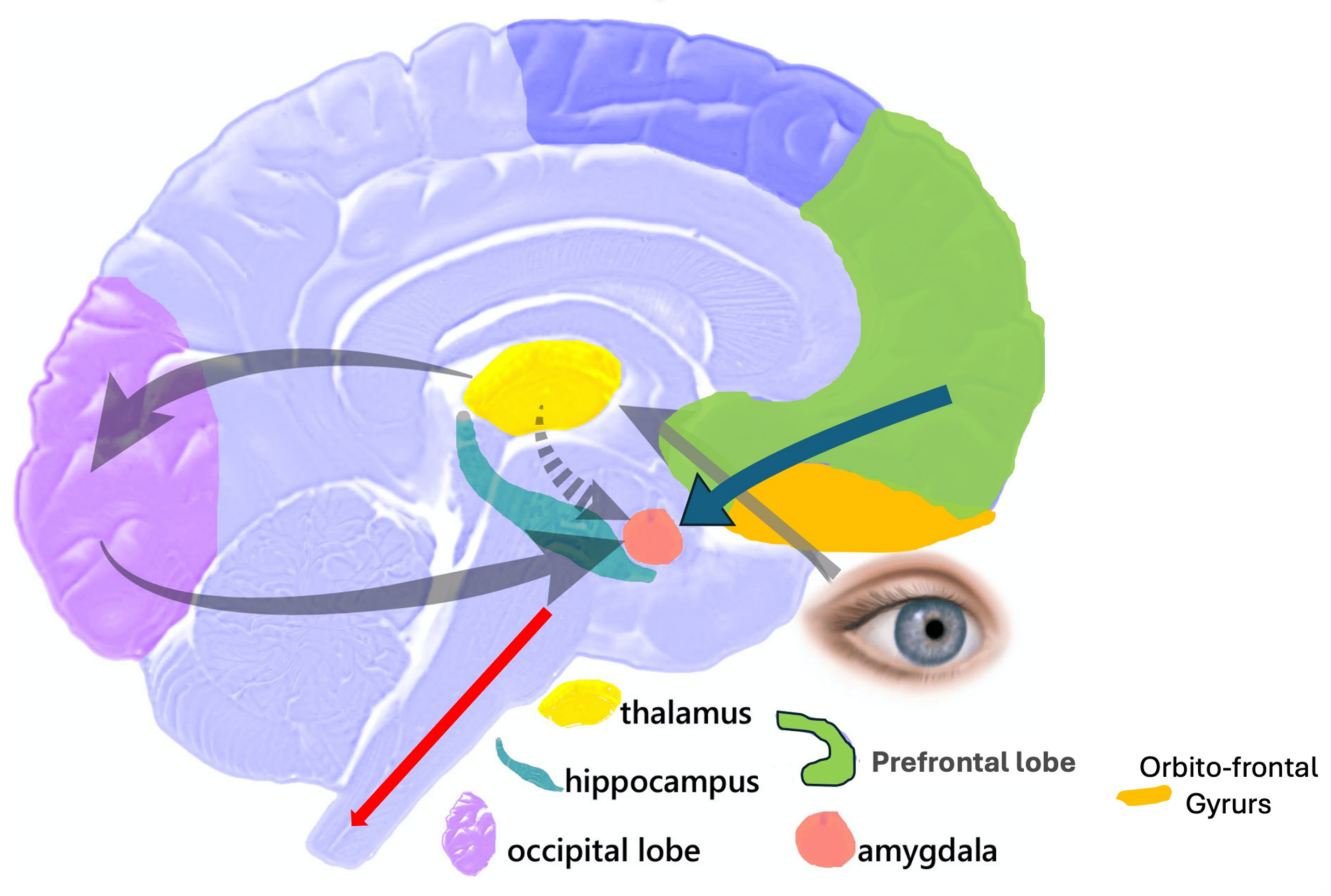
A schematic diagram of a neural network involved in the amygdala (emotional hijack). a) Threat stimulus from the eye (sensory organs) is transmitted to the thalamus and then loops through the visual cortex for perception (solid gray arrows). b) The information received in the thalamus is short-circuited to the amygdala (the emotional brain) for “fight-and-flight” readiness (interrupted gray arrow). At the same time, the adjacent hippocampus provides contextual information about the threat from the memory repertoire. c) Information from the thalamus is also processed through the prefrontal cortex (the rational brain) to make sense of the threat and then fed back to the amygdala to modulate accordingly. d) The hypothalamic-pituitary-adrenal axis processes the autonomic (sympathetic) response as per threat information from neural connections from the amygdala nearby. e) (Reproduced from Wikimedia Commons contributors. EQbrain optical stim en [Internet] under the Creative Commons Non-Commercial License, CC BY 4.0) Source: https://commons.wikimedia.org/wiki/File:EQbrain_optical_stim_en.jpg [97]. The image has been modified and relabeled for additional information. No built-in AI tools in any software, or other generative AI tools, were used to create/modify the figure.

A public example of this phenomenon occurred in 2018, when Turner [91], then a Port Authority commissioner, was filmed berating police officers during a traffic stop involving her daughter. Despite her professional stature, Turner’s emotionally reactive behavior and demands for preferential treatment illustrated how the amygdala can override prefrontal regulation, resulting in impaired judgment and socially inappropriate responses [92,93]. In contrast, effective emotional regulation recruits the vmPFC, dlPFC, and ACC, which modulate limbic activity through cognitive appraisal and the inhibition of impulsive reactions [94-96]. The vmPFC integrates emotional meaning and informs value-based decisions, while the ACC monitors emotional conflict and allocates attention to resolve it (Figure 1) [97].

A personal account from author Justin Bariso exemplifies this regulatory mechanism. After snapping at his children following a stressful day, Bariso recognized his overreaction and reflected on how increased self-awareness might prevent similar incidents in the future [98]. This capacity for emotional self-monitoring is mediated by prefrontal-limbic circuitry that supports reflective pauses, reappraisal, and behavior adjustment [99]. The autonomic nervous system (ANS), particularly its sympathetic and parasympathetic branches, governs the physiological expression of emotion. In high-stakes or emotionally charged contexts, the sympathetic branch initiates a “fight or flight” response, characterized by an elevated heart rate, pupil dilation, and the release of cortisol. Prolonged sympathetic activation, especially without cortical regulation, can impair both emotional balance and decision-making [100]. EI-enhancing interventions often aim to strengthen these top-down regulatory pathways. Mindfulness meditation, for instance, has been shown to improve prefrontal-limbic connectivity and decrease amygdala reactivity. Complementary strategies, such as deep breathing and cognitive reappraisal, can help downregulate physiological arousal and promote emotional stability [101].

In summary, the neurophysiology of EI involves the dynamic coordination of emotional sensing (amygdala), contextual memory (hippocampus), cognitive control (PFC, ACC), and social-emotional processing (insula and mirror neuron systems). Dysregulation, such as during an amygdala hijack, can lead to impulsive, emotionally charged decisions with social and personal consequences. Conversely, well-regulated neural systems enable empathy, resilience, and adaptive responses in complex interpersonal environments.

Current application and prospects

Emotional intelligence has become an increasingly important focus across diverse real-world domains, including education, healthcare, leadership, and mental health. Its proven impact on decision-making, interpersonal relationships, and adaptive functioning has led to growing implementation in practical settings and innovations to enhance EI across populations [102]. In education, EI is now recognized as foundational for academic success and emotional development. Social-emotional learning (SEL) practices that incorporate EI training have improved students' self-awareness, empathy, emotional regulation, and academic performance [103]. The Collaborative for Academic, Social, and Emotional Learning [104] has led initiatives globally to integrate EI into school curricula, promoting positive behavior and reducing emotional distress among students. Healthcare is another critical sector where EI plays a transformative role. Among physicians, nurses, and allied health professionals, higher EI is associated with better patient communication, lower burnout rates, enhanced diagnostic accuracy, and improved patient safety [105]. Empathy, a core EI domain, fosters stronger therapeutic alliances and improves patient outcomes [106]. As a result, medical schools and hospitals are increasingly incorporating EI training into medical education and professional development programs [107].

In organizational and leadership contexts, EI is strongly linked to transformational leadership, employee engagement, and team cohesion. Leaders with high EI are better equipped to manage workplace stress, resolve conflict, and inspire collaboration. Corporations are adopting EI assessments and training programs to enhance leadership pipelines, improve employee well-being, and boost productivity [108]. Technologically, integrating EI into artificial intelligence (AI) and human-computer interaction is an emerging frontier. Affective computing aims to equip machines to recognize, interpret, and respond to human emotions, enabling more intuitive interfaces and personalized user experiences [109]. Applications range from emotionally adaptive chatbots to therapeutic virtual agents in digital mental health platforms [110]. Looking ahead, the prospects for EI research and application are expansive. Neuroscientific tools, such as real-time fMRI, EEG, and machine learning algorithms, are enabling a deeper mapping of the neural correlates of EI, potentially leading to personalized cognitive-emotional training interventions [111].

Moreover, as global focus on mental health issues increases, EI-based curricula may be embedded not only in schools but also in workplaces and community health models to bolster emotional resilience and reduce the burden of affective disorders [112]. Policymakers are also beginning to recognize the societal value of EI. Public health campaigns and national education standards increasingly advocate for the inclusion of emotional literacy and resilience-building practices in educational settings [113]. These shifts recognize that EI is not only a personal asset but also a critical public good that enhances civic empathy, cooperation, and overall well-being [114].

Strengths and limitations

This narrative review offers an integrative, multidisciplinary synthesis of the neuroscience of EI, drawing on genetics, neuroanatomy, histology, neurophysiology, and neurotransmitter systems. Its primary strength lies in integrating diverse evidence streams into a cohesive neurobiological framework that emphasizes both innate and plastic elements of EI. The inclusion of emerging domains such as epigenetics, large-scale brain networks, and translational applications in education and healthcare enhances the depth and relevance of the review. Furthermore, it bridges theoretical constructs with practical implications, thereby advancing the conceptualization of EI as a biologically grounded and developmentally dynamic construct.

However, the narrative structure has inherent limitations. The lack of systematic inclusion criteria can lead to selection bias, and the absence of meta-analytic rigor limits the ability to assess effect sizes quantitatively and accurately. Differences in how EI is defined and measured across studies make comparisons difficult. Notably, inconsistencies in EI measurement tools and conceptual overlap with personality constructs may further confound interpretation. Much of the cited evidence remains correlational, limiting causal inference and generalizability. Additionally, while the review discusses implications for practice, it does not critically assess the empirical effectiveness of EI-based interventions. Future research should adopt standardized operational definitions and use systematic, longitudinal methodologies to improve causal understanding and strengthen validity.

## Conclusions

This review establishes EI as a biologically grounded, dynamic neurocognitive construct shaped by interactions among neural circuitry, genetic factors, and environmental plasticity. Integrating evidence from neuroimaging, genetics, and neurophysiology, it highlights multilevel mechanisms underlying emotional processing, regulation, and social adaptation. Framing EI within contemporary neuroscience enhances conceptual clarity and supports its translational relevance across education, healthcare, and organizational contexts. Future research should adopt longitudinal, mechanistic, and neurodevelopmentally informed approaches to refine assessment models and guide targeted, context-sensitive interventions that strengthen adaptive emotional functioning. 

## Appendices

Appendix A

Search Strategy

This search strategy was developed to support the narrative review titled 'Neuroscience of Emotional Intelligence: An Integrative Narrative Review'. It incorporates MeSH terms, controlled vocabulary, and relevant scholarly keywords across multiple databases.

1. Medical Subject Headings (MeSH)

Emotional Intelligence [MeSH] Neurosciences [MeSH] Brain [MeSH] Neuroimaging [MeSH] Genes [MeSH] Epigenesis, Genetic [MeSH] Neurotransmitter Agents [MeSH] Neural Pathways [MeSH] Social Behavior [MeSH] Prefrontal Cortex [MeSH] Amygdala [MeSH] Cerebral Cortex [MeSH]

2. Keywords and Synonyms

Emotional Intelligence

emotional intelligence, EI, trait emotional intelligence, ability emotional intelligence, emotional competence, affective intelligence

Neuroscience Terms

neuroscience, neurobiology, neurophysiology, neuroanatomy, neural architecture, neural networks, brain circuits, brain structure

Functional and Structural Brain Terms

prefrontal cortex, anterior cingulate cortex, amygdala, insula, hippocampus, limbic system, neural connectivity, white matter tracts, fMRI, DTI, EEG

Molecular and Genetic Terms

genetics, gene expression, polymorphism, serotonin transporter (5-HTTLPR), COMT, DRD4, oxytocin receptor (OXTR), dopamine, glutamate, GABA, epigenetics, DNA methylation, histone modification

Application and Contextual Keywords

social cognition, emotional regulation, empathy, executive function, stress response, resilience, decision-making, mindfulness, social-emotional learning, affective computing

3. Boolean Search Syntax (Example for PubMed)

("Emotional Intelligence"[MeSH Terms] OR "emotional intelligence" OR "EI" OR "emotional competence") AND ("Neurosciences"[MeSH Terms] OR neuroscience OR neurobiology OR "brain structure" OR "neural circuits") AND ("Neuroimaging"[MeSH Terms] OR fMRI OR DTI OR EEG OR "functional connectivity") AND ("Genetics"[MeSH Terms] OR genes OR polymorphism OR epigenetics OR "DNA methylation" OR "5-HTTLPR" OR "COMT") AND ("Prefrontal Cortex"[MeSH Terms] OR amygdala OR hippocampus OR "anterior cingulate cortex" OR insula OR "limbic system")

4. Database-Specific Strategy Notes

Database

Strategy Notes

PubMed

Used MeSH terms + keywords; apply filters for review articles, humans, and age range.

PsycINFO

Used 'emotional intelligence' in title/abstract; combine with neuroscience terms.

Scopus/Web of Science

Used title/abstract/keyword fields; apply truncation (*), Boolean logic.

Embase

Used EMTREE terms; include emotional ability, neuroscience filters.

Appendix B

Inclusion Criteria

• Peer-reviewed empirical studies, systematic reviews, and meta-analyses published in English.

• Studies explicitly addressing emotional intelligence (EI) with relevance to neuroscience (e.g., neuroimaging, neurophysiology, neuroanatomy, neurochemistry, genetics, epigenetics).

• Evidence from both human and validated animal models is required to establish translational relevance to EI.

• Neuroscience-informed interventions targeting EI (e.g., mindfulness, cognitive reappraisal, social-emotional training).

• Articles published from the database inception to March 31, 2025.

Exclusion Criteria

• Articles not available in English or without full-text access.

• Theoretical papers lacking empirical or neuroscientific data.

• Studies exclusively focused on general intelligence, IQ, or personality traits without specific reference to EI.

• Clinical studies examining emotional dysregulation in psychiatric disorders without reference to EI constructs.

• Papers limited to behavioral or social outcomes without neurobiological analysis or relevance.

• Non-peer-reviewed articles, commentaries, book chapters, editorials, or conference abstracts.
